# Dataset on the soils of medieval archaeological monuments in the forest-steppe zone of the East European plain

**DOI:** 10.1016/j.dib.2020.105555

**Published:** 2020-04-20

**Authors:** Fatima Kurbanova, Tatiana Puzanova, Olga Rudenko, Gennadiy Starodubtsev

**Affiliations:** a Faculty of Soil Science, Lomonosov Moscow State University, Leninskie Gory, MSU, 119991 Moscow, Russia; b Faculty of Geography, Lomonosov Moscow State University, Leninskie Gory, MSU, 119991 Moscow, Russia; c Orel State University, 95 Komsomolskaya str., 302026 Orel, Russia; d Kursk State Regional Museum of Archaeology, 6 Pionerov str., 305001 Kursk, Russia

**Keywords:** Paleosols, Holocene, Spore-pollen, Paleolandscape, Archaeological monuments, Paleoclimate, Non-pollen palynomorphs, Geoarchaeology

## Abstract

One of the natural archives that can save information about the environmental conditions of the past is soils buried under embankments of burial complexes. Due to isolation from external environmental factors soils retain information about the features of the natural environment at the time of its burial. In this work we present a dataset on soils buried under four mounds in the Middle Ages. The soils were buried under mounds in a short time interval – 25–50 years. For comparison, the data on the surface soil located near the barrows are also presented. Obtained dataset includes detailed morphological field description of the soils and their physico-chemical analysis, such as granulometry, elemental analysis, fractions of iron and selected chemical data. Obtained data can be used to identify the dynamics of forest-steppe landscapes in the XIth century. The Medieval Warm Period and the subsequent humidisation of the climate over a short time interval had a significant impact on natural conditions and the migration of the population of the steppes of Eurasia. A comparative analysis of the properties of soils buried under archaeological sites of different ages allows examining in details the changes in the natural environment and its components over time. Moreover, soils are capable of storing a whole range of additional features of non-pedogenic origin that can be used for a more detailed reconstruction of the natural environment. The data on spores, pollen and non-pollen palynomorphs of the soil profiles are also presented in this article.

Specifications table

**Table:** 

Subject	Soil Science, Earth Science
Specific subject area	Paleopedology, Palynology, Paleoclimate, Paleoenvironmental reconstruction, Geoarchaeology
Type of data	Tables, images, graphs
How data were acquired	Soil horizons were described according to FAO Guidelines for Soil Description [1], soil colour was determined in the field using the Munsell Soil Colour Charts [2], field identification of soils was performed according to the WRB [3]. Laboratory analyses were performed on samples taken equidistantly (10 cm down to 1 m and 20 cm below 1 m). The elemental analysis was performed using the X-ray fluorescence spectrometry method after loss on ignition determination (1000 C) using the Philips PW2400 Sequential WXRF Spectrometer (Malvern Panalytical, Almelo, The Netherlands). Two methods to determine the granulometric composition of soils were used: pipette method, in which soil is dispersed by treating with a solution of sodium pyrophosphate (Na_4_P_6_O_18_) and laser granulometry method on the device “Analysette 22. Laser Klasse 1. Fritsch”*.* The dithionite and oxalate extractable fractions of iron were acquired according to Mehra and Jackson [4] and analysed using a Cary 60 Spectrophotometer (Agilent Technologies, the USA); the total carbonate content was determined on the base of the destruction of CaCO_3_ by acid and subsequent precipitation of the carbonate ion [5]; рН in water suspension (soil: water ratio 1:2.5) were analysed using standard methods [6]. C/N ratio were obtained using CHNS Elementar Analysensysteme GmbH VARIO EL III V4.01 20.Aug. 2002 (Germany). The taxonomic identification of microfossils was carried out using keys and atlases, as well as electronic databases of photo pollen and non-pollen palynomorphs (NPP database, Paldat, European pollen database, etc.) with a use of a Motic-B1- microscope 220A at magnification × 400. To calculate percentage ratios and build spore-pollen diagrams, the Tilia / TiliaGraph / TGView software package was used.
Data format	Raw
Parameters for data collection	Samples were taken from buried and surface soils. Four buried and one surface soil are located in the watershed surface, under the one forestland in Kursk region. Three soils were buried in the second quarter/middle of XIth century, one in the second half of XIth century. The thickness of mounds is 40–60 cm.
Description of data collection	A total of 63 soil samples (500–700 g) were collected from the five pits. Samples were taken from every 10 cm (first metre) and 20 cm (second metre) but taking into account the boundaries of the horizons. Samples for spore-pollen analysis were collected from upper 5 cm of the soils.
Data source location	Buried and surface soils were formed on the right bank of the river Psel near settlement Gochevo, Belovsky district, Kursk region, Russia.
	GPS coordinates for collected samples:
	1f-19. 51°08′42.6″N 35°52′35.9″E
	2b-19. 51°08′43.4″N 35°52′37.6″E
	3b-19. 51°08′43.1″N 35°52′35.7″E
	4b-19. 51^о^08′42.0″N 35^о^52′40.0″E
	5b-19. 51°08′42.2″N 35°52′40.3″E
Data accessibility	Data are with this article

## 1 Value of the data

• Data contained can be used for paleoclimatic reconstrutions of the second half of the Holocene in the forest-steppe zone.
• Data may be useful for researches who studying soil genesis and evolution.
• Data provides wide range of analysis of the surface and certain buried soils in XI century AD.
• Data could be useful for palynologists to supplement existing spore-pollen data for the Medieval time period.
• Data on archaeological objects obtained by a combination of various natural-scientific methods can give the ideas about the migration of ancient ethnic groups.

## 1 Data description

**Fig. 1:**
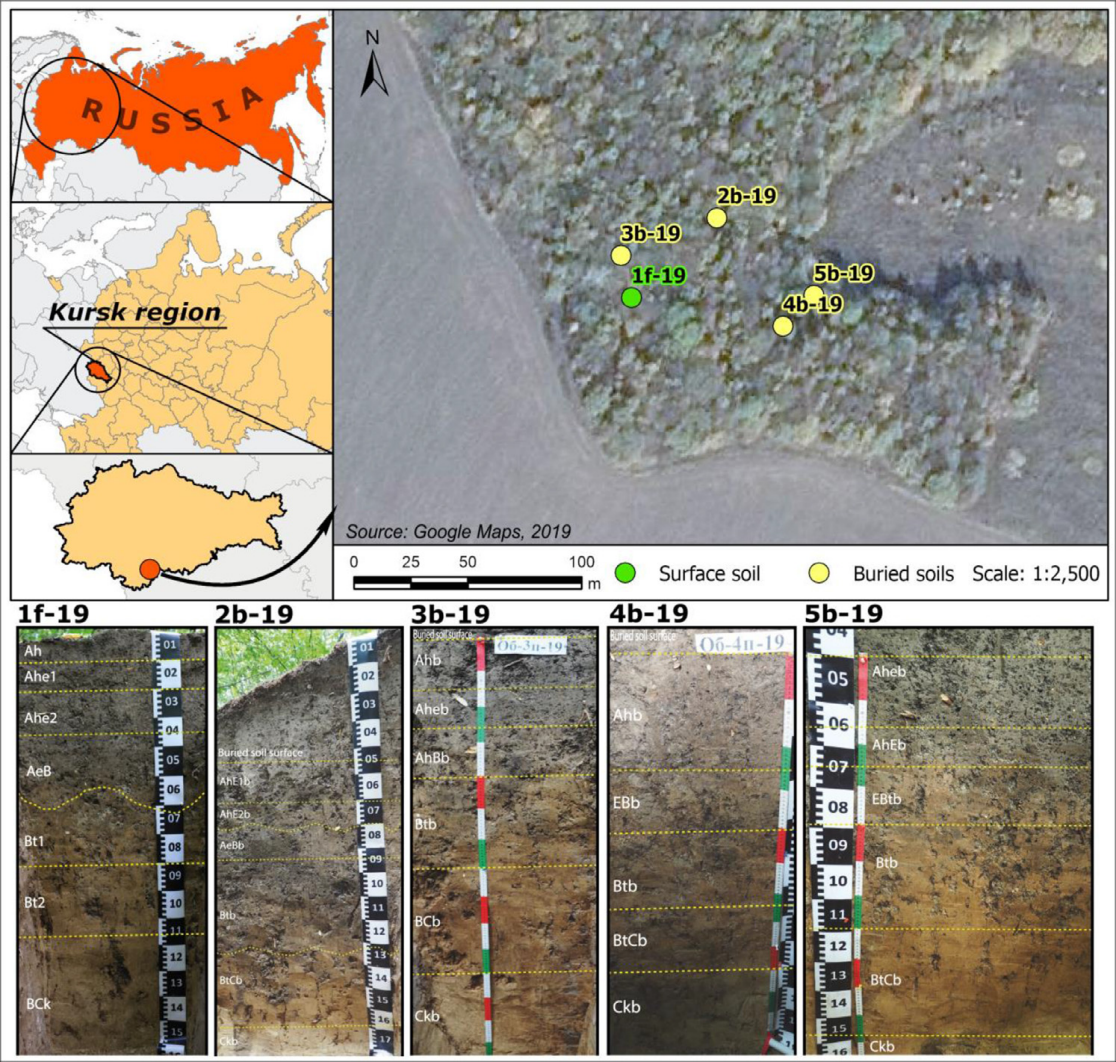
Location of the dataset area and profiles of buried and surface soils.

Fig. 1 shows location of the dataset area and soil images with boundaries and indexes of the horizons according to the FAO Guidelines for Soil Description [1].

Morphological field description is presented in Table 1 and includes information about indexes and depth of horizons, colour of general matrix in moist conditions, size, colour and abundance of clay coatings, type of a soil structure, abundance of roots and type of horizon boundary (topography and distinctness) of each soil.

**Table 1:** Field description of the soils*.

Horizon	Depth, Cm⁎⁎	Colour of general matrix (moist)	Coatings	Colour of coatings (moist)	Structure	Abundance of roots	Horizon boundary	
							Topography	Distinctness, cm
**Surface soil 1f-19 -***Greyzemic Luvic Phaeozem Cutanic*								
Ah	0–10	7,5 YR 4–5/1	**–**	**–**	WE / GR	Many	Wavy	Gradual
Ahe1	10–20	7,5 YR 5–6/1	**–**	**–**	MO / SB GR PL	Common	Wavy	Gradual
Ahe2	20–36	7,5 YR 4/1	**–**	**–**	MO / AB	Common	Wavy	Gradual
AeB	36–50(64)	7,5 YR 3/2	Medium-F	5 YR 3/2	MO / AB	Few	Wavy	Clear
Bt1	50(64)−90	10 YR 5/4	Medium-C	5 YR 3/2	MO / PR AB	Few	Wavy	Gradual
Bt2	90–105	10 YR 5/4	Thick-A	5 YR 3/2	MO / PR	Few	Wavy	Clear
BCk	105–150	10 YR 6/4	Thick-C	5 YR 3/2	MO / AB PR	Single	**–**	**–**
**Buried soil 2b-19 -***Greyzemic Luvic Phaeozem Cutanic*								
AhEb1	0–16	10 YR 5–4/1	**–**	**–**	MO / AB	Few	Wavy	Gradual
AhEb2	16–26(30)	10 YR 3/1	**–**	**–**	MO / AB	Few	Wavy	Gradual
AeBb	26(30)−40	10 YR 4/2	Thin-F	**–**	MO / AB	Few	Wavy	Gradual
Btb	40–80(84)	7,5 YR 5/4	Medium-A	5 YR 3/2	MO / PR AB	Few	Wavy	Gradual
BtCb	80(84)−110	7,5 YR 3/2	Thick-A	5 YR 3/2	WM / SB	Few	Wavy	Clear
Сkb	110–130	10 YR 5/6	**–**	**–**	WE / AB	Few	**–**	**–**
**Buried soil 3b-19 -***Greyzemic Luvic Phaeozem Cutanic*								
Ahb	0–16	10 YR 3/1	**–**	**–**	WM / SB	Many	Wavy	Gradual
Aheb	16–26	10 YR 2/1	**–**	**–**	WM / SB	Many	Wavy	Gradual
AhBb	26–40	7,5 YR 3/2	**–**	**–**	MO / AB	Common	Wavy	Gradual
Btb	40–70	10 YR 5/4	Medium-A	5 YR 3/2	MO / AB	Common	Wavy	Gradual
BCb	70–110(120)	10 YR 5/6	Medium -A	10 YR 4–3/2	MO / PR	Common	Wavy	Clear
Сkb	110(120)−150	10 YR 5/6	**–**	**–**	WE / SB	Few	**–**	**–**
**Buried soil 4b-19 -***Greyzemic Luvic Phaeozem Cutanic*								
Ahb	0–26	10 YR 3/1	**–**	**–**	WM / PR	Many	Wavy	Gradual
EBb	26–40	10 YR 3–4/1	**–**	**–**	WM / SB	Many	Wavy	Gradual
Btb	40–70	10 YR 4/4	Medium-A	**–**	MO / AB	Common	Wavy	Gradual
BtCb	70–90(100)	10 YR 5/6	Medium-A	10 YR 4/4	MO / PR	Common	Wavy	Clear
Сkb	90(100)−130	10 YR 6–7/4	**–**	**–**	WE / SB	Few	**–**	**–**
**Buried soil 5b-19**								
Aheb	0–15	10 YR 3/1	**–**	**–**	WM / AB	Many	Wavy	Gradual
AhEb	15–26	10 YR 3–2/1	**–**	**–**	WM / SB	Common	Wavy	Gradual
EBtb	26–40	10 YR 4/2	Medium-C	10 YR 4/3	MO / AB	Common	Wavy	Gradual
Btb	40–70	10 YR 5/4	Medium-A	10 YR 4/3 10 YR 6–7/2	WM / PR	Common	Wavy	Gradual
BtCb	70–110	10 YR 5/4	Medium-A	10 YR 4/4	WM / PR	Few	Wavy	Clear
Сkb	110–120	10 YR 6–7/4	**–**	**–**	WE / SB	Few	**–**	**–**

⁎ Indexes are based on FAO guide for soil description (2006).

⁎⁎ For the upper horizons: in the numerator – depth in the control section; in the denominator - mean depth, in bold, and the data spread, in parentheses. **Colour of general matrix (moist), Colour of coatings (moist)**: according to Munsell soil colour charts (1994); **Coatings:**Thickness: medium (0.5–1), thick>1; Abundance: F - few, C - common, A – abundant; **Structure**: Grades: WE – Weak, MO – Moderate, WM – Weak to Moderate, ST – Strong; Types: AB – Angular blocky, GR – Granular, SB – Subangular blocky, PL – Platy, PR – Prismatic.

Tables 2 and 3 show the results of XRF analysis. Total concentrations of major oxides (Na_2_O, MgO, Al_2_O_3_, SiO_2_, K_2_O, CaO, TiO_2_, MnO, Fe_2_O_3_, P_2_O_5_) and loss on ignition (LOI) in the buried and surface soils are presented in Table 2. Table 3 includes data on total concentrations of trace elements: Cr, V, Co, Ni, Cu, Zn, Rb, Sr, Zr, Ba, Y, Nb, Pb. Table 4 contains data on selected chemical features, including pH, content of carbon and nitrogen, C/N relation, amount of calcium carbonate (%) and oxalate extractable fractions of iron. Data on particle size distribution made by pipette method reflected in Table 5. The boundaries between six particle size classes were defined in accordance with the Russian conventional fraction groups (0,25–1; 0,05–0,25; 0,01–0,05; 0,005–0,01; 0,001–0,005; <0,001 mm). Table 6 contains data on grain size distribution done by laser granulometry. Table 7 includes results of spore-pollen analysis and provides data on spores, pollen and non-pollen palynomorphs.

**Table 2:** Total concentrations of major elements and loss on ignition (LOI) in the buried and surface soils, %.

Horizon	Depth, cm	LOI	Na2O	MgO	Al2O3	SiO2	K2O	CaO	TiO2	MnO	Fe2O3	P2O5
**Surface soil 1f-19**												
Аh	0–10	12,13	0,78	0,67	7,12	72,25	2,17	1,16	0,76	0,119	2,33	0,15
Ahe1	10–20	6,34	0,89	0,66	7,70	77,80	2,21	0,80	0,78	0,096	2,32	0,09
Ahe2	20–30	6,09	0,91	0,68	7,92	77,68	2,23	0,80	0,78	0,083	2,45	0,09
AeB	40–50	6,33	0,78	0,87	9,03	75,34	2,33	0,86	0,79	0,059	3,21	0,09
Bt1	60–70	6,29	0,79	0,87	9,08	75,44	2,27	0,78	0,79	0,042	3,23	0,10
	80–90	7,61	0,75	1,00	9,88	72,46	2,23	0,81	0,82	0,045	3,99	0,11
Bt2	90–100	7,70	0,73	1,06	10,27	71,67	2,21	0,85	0,85	0,052	4,22	0,11
BCk	100–120	7,99	0,71	1,05	10,18	71,49	2,09	0,97	0,83	0,075	4,21	0,10
	140–150	13,53	0,45	0,93	8,77	60,16	1,71	9,62	0,78	0,093	3,57	0,11
**Buried soil 2b-19**												
AhEb1	0–10	6,00	0,89	0,72	8,29	76,98	2,27	0,83	0,80	0,112	2,73	0,08
AhEb2	20–30	6,02	0,87	0,77	8,49	76,50	2,28	0,81	0,81	0,117	2,94	0,09
AeBb	30–40	7,27	0,77	0,94	9,53	73,51	2,33	0,91	0,80	0,067	3,49	0,10
Btb	40–50	7,65	0,73	0,95	9,49	73,15	2,27	0,90	0,79	0,050	3,63	0,10
	60–70	7,20	0,74	0,94	9,45	73,41	2,30	0,87	0,81	0,049	3,81	0,11
	70–80	7,14	0,80	1,02	9,79	73,10	2,28	0,94	0,80	0,055	3,67	0,11
BtCb	80–90	7,36	0,80	1,04	9,85	72,71	2,24	0,92	0,81	0,055	3,79	0,12
	100–110	7,12	0,74	0,90	9,09	73,85	2,15	1,51	0,79	0,051	3,39	0,11
Ckb	110–120	10,06	0,59	0,79	7,42	67,75	1,91	7,76	0,73	0,043	2,55	0,13
	120–140	9,69	0,61	0,82	7,78	68,98	1,91	6,40	0,74	0,051	2,63	0,11
**Buried soil 3b-19**												
Ahb	0–10	6,73	0,78	0,86	9,14	74,57	2,42	0,94	0,81	0,082	3,25	0,10
Aheb	20–30	6,56	0,75	0,91	9,42	74,24	2,37	0,98	0,79	0,053	3,51	0,10
AhBb	30–40	7,10	0,74	0,89	9,32	73,99	2,29	0,89	0,79	0,046	3,56	0,09
Btb	50–60	7,24	0,71	0,98	9,62	73,16	2,27	0,95	0,82	0,049	3,80	0,11
BCb	70–80	7,57	0,71	1,07	9,96	71,95	2,19	1,00	0,85	0,063	4,22	0,11
	90–100	8,30	0,57	1,04	10,46	70,67	2,02	1,03	0,85	0,083	4,59	0,10
	100–120	8,58	0,58	1,00	10,19	71,03	1,92	1,02	0,85	0,090	4,37	0,07
Ckb	120–140	8,20	0,67	0,99	9,75	71,59	1,99	1,59	0,83	0,071	3,95	0,08
	140–150	9,42	0,60	0,96	9,27	68,79	1,95	4,12	0,82	0,075	3,62	0,08
**Buried soil 4b-19**												
Ahb	0–10	6,39	0,83	0,85	9,02	75,22	2,43	0,90	0,80	0,060	3,10	0,10
	20–30	7,23	0,73	0,94	9,62	73,18	2,40	0,92	0,79	0,053	3,73	0,11
EBb	30–40	7,01	0,72	0,95	9,70	73,40	2,35	0,87	0,79	0,047	3,76	0,11
Btb	50–60	7,45	0,72	0,97	9,83	72,77	2,29	0,86	0,79	0,047	3,86	0,12
BtCb	70–80	7,70	0,71	1,05	10,10	71,73	2,24	0,90	0,82	0,054	4,29	0,11
	90–100	6,19	0,73	0,82	8,91	75,91	2,10	0,86	0,80	0,046	3,24	0,09
Сkb	100–120	6,72	0,73	0,84	9,04	75,04	2,14	0,92	0,81	0,048	3,32	0,10
	120–140	10,41	0,56	0,92	8,20	66,75	1,87	7,01	0,78	0,065	3,04	0,10
**Buried soil 5b-19**												
Aheb	0–10	7,03	0,79	0,84	8,93	74,46	2,44	0,93	0,82	0,076	3,26	0,10
AhEb	20–30	7,25	0,73	0,92	9,30	73,83	2,34	0,88	0,77	0,050	3,55	0,10
Ebtb	30–40	7,06	0,74	0,91	9,38	73,84	2,38	0,88	0,79	0,049	3,57	0,10
Btb	50–60	7,02	0,75	0,94	9,30	73,92	2,32	0,89	0,80	0,047	3,61	0,12
BtCb	70–80	7,41	0,76	1,04	9,64	72,50	2,33	0,96	0,82	0,053	4,03	0,13
	90–100	6,88	0,72	0,84	9,02	74,96	2,14	0,89	0,78	0,047	3,32	0,11
	100–120	7,58	0,68	0,78	8,13	73,31	2,09	3,50	0,74	0,043	2,73	0,11
Ckb	110–120	7,70	0,69	0,79	8,02	72,94	2,09	3,96	0,74	0,047	2,63	0,11

**Table 3:** Total concentrations of trace elements in the buried and surface soils, mg·kg^−1^.

Horizon	Depth, cm	Cr	V	Co	Ni	Cu	Zn	Rb	Sr	Zr	Ba	Y	Nb	Pb
**Surface soil 1f-19**														
Аh	0–10	77	74	<10	21	11	51	77	94	728	494	31	17	24
Ahe1	10–20	74	73	12	19	13	46	79	95	794	490	32	17	19
Ahe2	20–30	78	68	13	19	14	44	79	95	791	466	33	16	16
AeB	40–50	88	79	12	25	10	52	81	93	728	443	32	18	21
Bt1	60–70	82	93	17	25	14	53	78	91	771	458	35	18	18
	80–90	101	103	13	27	11	56	83	93	718	433	39	18	22
Bt2	90–100	101	106	12	29	12	61	83	93	721	431	39	18	26
BCk	100–120	107	105	17	31	13	60	83	91	703	449	37	18	24
	140–150	103	81	11	37	15	55	74	128	456	388	35	17	16
**Buried soil 2b-19**														
AhEb1	0–10	73	79	13	25	17	53	82	95	786	508	31	17	17
AhEb2	20–30	83	77	10	25	16	54	83	95	768	494	35	18	21
AeBb	30–40	91	94	12	25	12	54	83	94	708	471	34	17	20
Btb	40–50	92	88	<10	29	12	53	82	93	720	447	35	17	22
	60–70	90	94	14	26	10	51	81	92	709	442	39	18	19
	70–80	104	96	<10	29	11	55	82	97	736	431	37	17	12
BtCb	80–90	90	105	14	28	10	58	83	97	728	475	39	18	27
	100–110	83	85	16	30	<10	50	77	99	723	428	36	16	20
Ckb	110–120	75	73	<10	22	<10	42	68	137	619	398	32	16	15
	120–140	66	70	10	26	<10	42	69	129	652	412	31	17	19
**Buried soil 3b-19**														
Ahb	0–10	88	79	10	25	16	52	85	97	697	469	38	18	18
Aheb	20–30	96	87	12	24	<10	52	81	95	701	468	36	16	22
AhBb	30–40	92	90	14	24	<10	50	80	93	725	427	34	17	19
Btb	50–60	97	91	15	26	<10	53	82	94	725	446	41	19	22
BCb	70–80	92	106	14	32	14	56	80	96	690	424	41	18	26
	90–100	107	112	18	38	16	59	83	87	648	430	43	19	25
	100–120	107	113	17	41	17	59	82	86	656	457	41	19	28
Ckb	120–140	95	99	14	34	14	54	80	95	664	444	38	19	21
	140–150	88	100	13	31	13	52	76	110	627	445	35	18	20
**Buried soil 4b-19**														
Ahb	0–10	88	79	13	25	15	48	84	97	717	477	36	16	20
	20–30	89	86	13	28	11	52	85	94	688	467	37	17	22
EBb	30–40	89	85	13	23	<10	53	83	91	720	492	34	18	20
Btb	50–60	81	95	<10	26	<10	56	83	90	733	478	39	17	21
BtCb	70–80	93	104	17	28	11	58	82	91	679	408	38	17	23
	90–100	87	85	11	25	11	47	79	88	799	452	36	18	19
Ckb	100–120	81	86	<10	27	<10	50	78	89	795	432	43	17	23
	120–140	79	79	<10	28	13	43	73	130	607	390	31	17	17
**Buried soil 5b-19**														
Aheb	0–10	86	83	10	26	13	53	84	95	715	472	36	16	20
AhEb	20–30	87	86	<10	24	<10	52	82	92	733	460	36	16	22
EBtb	30–40	90	86	14	25	<10	51	83	92	727	447	36	16	20
Btb	50–60	85	96	16	25	<10	53	81	93	741	442	35	17	22
BtCb	70–80	93	102	12	33	12	55	83	96	689	451	37	18	22
	90–100	86	95	15	25	11	49	77	88	740	423	34	17	21
	100–110	71	69	<10	26	10	43	72	102	707	415	40	16	16
Ckb	110–120	79	71	10	25	10	42	72	108	683	398	32	16	18

**Table 4:** Selected chemical features in the surface and buried soils.

Horizon	Depth, cm	pH	N, %	С, %	C/N	CaCO_3,_ %	Fed, %*	Feo, %
**Surface soil**								
Аh	0–10	6,54	0,7	4,6	6,8	–	0,19	0,73
Ahe1	10–20	5,98	0,3	1,7	5,4	–	0,17	0,8
Ahe2	20–30	6,34	0,2	1,3	6,1	–	0,16	0,79
	30–40	6,14	0,3	1,4	4,7	–	–	–
AeB	40–50	5,96	0,2	1,1	5,0	–	0,15	1,07
Bt1	50–60	6,00	–	–	–	–	–	–
	60–70	5,95	–	–	–	–	0,13	0,96
	70–80	5,77	–	–	–	–	–	–
	80–90	5,64	–	–	–	–	0,13	1,14
Bt2	90–100	5,68	–	–	–	–	0,14	1,23
BCk	100–120	5,87	–	–	–	0,18	0,13	1,33
	120–140	8,12	–	–	–	2,20	–	–
	140–150	8,20	–	–	–	3,61	0,07	0,92
**Buried soil 2b-19**								
AhEb1	0–10	6,12	0,2	1,1	5,1	–	0,17	0,81
	10–20	6,00	0,2	1,0	4,5	–	–	–
AhEb2	20–30	6,02	0,3	1,2	4,2	–	0,16	0,91
AeBb	30–40	6,40	0,3	1,2	4,1	–	0,12	1,02
Btb	40–50	6,07	–	–	–	–	0,12	1,05
	50–60	6,23	–	–	–	–	–	–
	60–70	5,97	–	–	–	–	0,13	1,05
	70–80	6,37	–	–	–	–	0,12	1,02
BtCb	80–90	6,18	–	–	–	–	0,12	1,11
	90–100	6,43	–	–	–	–	–	–
	100–110	7,56	–	–	–	–	0,09	0,98
Ckb	110–120	8,29	–	–	–	2,55	0,05	0,70
	120–140	8,34	–	–	–	2,55	0,05	0,72
**Buried soil 3b-19**								
Ahb	0–10	6,16	0,3	1,5	4,8	–	0,14	1,01
	10–20	6,49	0,3	1,3	4,6	–	–	–
Aheb	20–30	6,55	0,2	0,9	5,3	–	0,13	1,11
AhBb	30–40	6,45	0,4	1,2	3,0	–	0,13	1,07
Btb	40–50	6,89	–	–	–	–	–	–
	50–60	6,82	–	–	–	–	0,14	1,19
	60–70	6,91	–	–	–	–	–	–
BCb	70–80	7,07	–	–	–	–	0,13	1,27
	80–90	7,06	–	–	–	–	–	–
	90–100	7,02	–	–	–	–	0,13	1,45
	100–120	7,11	–	–	–	–	0,11	1,30
Ckb	120–140	8,23	–	–	–	0,18	0,09	1,20
	140–150	8,32	–	–	–	1,50	0,08	1,18
**Buried soil 4b-19**								
Ahb	0–10	5,87	0,3	1,3	4,8	–	0,10	1,1
	10–20	5,90	0,4	1,6	4,0	–	–	–
	20–30	6,18	0,3	1,3	3,9	–	0,12	1,03
EBb	30–40	5,97	0,3	1,0	3,7	–	0,12	1,2
Btb	40–50	6,04	–	–	–	–	–	–
	50–60	5,93	–	–	–	–	0,12	1,27
	60–70	5,71	–	–	–	–	–	–
BtCb	70–80	5,87	–	–	–	–	0,13	1,31
	80–90	6,10	–	–	–	–	–	–
	90–100	6,45	–	–	–	–	0,07	0,99
Ckb	100–120	7,89	–	–	–	0,18	0,06	0,9
	120–140	8,44	–	–	–	2,55	0,05	0,98
**Buried soil 5b-19**								
Aheb	0–10	6,15	0,3	1,7	5,0	–	0,13	1,15
	10–20	6,21	0,3	1,3	4,5	–		
AhEb	20–30	6,17	0,2	0,9	4,4	–	0,11	1,28
EBtb	30–40	6,31	0,1	0,7	5,3	–	0,11	1,17
Btb	40–50	6,13	–	–	–	–		
	50–60	6,08	–	–	–	–	0,11	1,17
	60–70	5,97	–	–	–	–		
BtCb	70–80	6,16	–	–	–	–	0,11	1,25
	80–90	6,10	–	–	–	–		
	90–100	5,83	–	–	–	–	0,07	0,99
	100–120	8,02	–	–	–	–	0,05	0,86
Ckb	110–120	8,31	–	–	–	2,02	0,04	0,79

⁎ Iron (Fe_2_O_3_) fractions: Fed – dithionite extractable (free) iron; Feo – oxalate extractable (active) iron.

**Table 5:** Grain size distribution, mm (Pipette method).

Horizon	Depth, cm	0,25–1	0,05–0,25	0,01–0,05	0,005–0,01	0,001–0,005	<0,001
**Surface soil**							
Аh	0–10	0,9	2,5	58,7	10,4	11,2	16,4
Ahe1	10–20	0,5	1,9	55,2	10,5	11,1	20,8
Ahe2	20–30	0,4	3,7	53,2	10,3	10,0	22,4
	30–40	0,4	3,4	53,7	7,2	9,0	26,4
AeB	40–50	0,4	3,0	50,7	7,9	9,5	28,5
Bt1	50–60	0,2	3,2	48,2	9,0	9,5	29,8
	60–70	0,3	6,7	46,9	8,6	8,7	28,8
	70–80	0,3	4,8	48,6	9,0	8,5	28,8
	80–90	0,3	3,9	44,0	8,9	8,7	34,3
Bt2	90–100	1,1	4,2	40,6	8,1	8,7	37,4
BCk	100–120	2,6	3,7	39,0	8,3	9,8	36,7
	120–140	3,2	7,4	33,0	8,3	14,4	33,8
	140–150	1,7	4,7	39,5	8,6	6,6	38,9
**Buried soil 2b-19**							
AhEb1	0–10	0,5	4,1	52,4	8,9	9,3	24,8
	10–20	0,5	3,5	52,0	9,0	9,7	25,2
AhEb2	20–30	0,6	4,6	50,1	10,0	8,9	25,7
AeBb	30–40	0,6	5,8	49,6	6,5	7,3	30,2
Btb	40–50	0,6	4,3	47,0	7,2	9,1	31,9
	50–60	0,4	4,0	46,4	8,8	8,9	31,5
	60–70	0,4	5,6	44,3	8,9	8,9	30,5
	70–80	0,3	3,0	43,1	7,8	9,3	32,7
BtCb	80–90	0,3	2,9	45,4	9,5	8,5	33,4
	90–100	0,7	3,6	46,6	8,5	8,7	31,9
	100–110	0,4	3,7	50,2	7,8	8,0	29,9
Ckb	110–120	0,3	4,8	49,2	7,6	11,3	26,8
	120–140	2,0	4,1	51,2	6,7	10,4	25,6
**Buried soil 3b-19**							
Ahb	0–10	0,3	3,0	51,0	9,7	9,9	26,2
	10–20	0,3	2,2	50,2	10,0	8,8	28,5
Aheb	20–30	0,3	2,1	50,1	10,3	8,3	29,0
AhBb	30–40	0,4	5,8	49,6	6,7	7,0	30,6
Btb	40–50	0,3	5,3	47,0	7,4	7,8	32,2
	50–60	0,3	5,5	45,7	9,4	8,8	30,3
	60–70	0,3	4,2	43,0	9,7	9,4	33,4
BCb	70–80	0,7	4,2	42,4	7,7	9,7	35,3
	80–90	1,6	4,1	41,3	7,4	8,8	36,9
	90–100	2,8	5,8	37,1	7,2	8,2	38,9
	100–120	3,6	6,1	35,4	8,1	9,2	37,6
Ckb	120–140	2,2	7,3	38,4	7,8	13,1	31,2
	140–150	1,6	6,8	41,1	8,9	11,1	30,5
**Buried soil 4b-19**							
Ahb	0–10	0,25	5,99	51,79	9,25	9,50	23,22
	10–20	0,22	5,11	50,70	8,23	9,25	26,49
	20–30	0,20	4,09	47,96	9,72	8,44	29,59
EBb	30–40	0,16	6,07	46,95	9,88	9,17	27,77
Btb	40–50	0,22	7,78	47,04	6,86	7,90	30,20
	50–60	0,17	6,50	45,39	7,76	8,33	31,85
	60–70	0,26	6,94	45,59	7,98	8,93	30,30
BtCb	70–80	0,48	5,07	41,73	8,80	9,06	43,86
	80–90	0,61	5,11	47,26	7,92	9,05	34,57
	90–100	1,86	6,51	50,00	6,92	6,42	28,29
Ckb	100–120	1,42	5,31	51,16	7,22	5,89	29
	120–140	2,51	8,06	42,54	6,92	9,40	30,57
**Buried soil 5b-19**							
Aheb	0–10	0,25	5,03	50,86	8,74	10,06	25,06
	10–20	0,30	3,78	50,11	8,27	8,60	28,94
AhEb	20–30	0,29	3,93	48,74	9,38	7,63	30,03
EBtb	30–40	0,24	5,67	46,90	9,93	8,02	29,24
Btb	40–50	0,26	5,84	47,03	6,45	7,95	32,47
	50–60	0,22	3,17	44,44	9,86	8,36	32,83
	60–70	0,25	7,09	40,87	8,88	9,54	33,37
BtCb	70–80	0,36	4,22	43,10	10,14	8,97	33,21
	80–90	0,83	4,78	43,25	8,06	9,39	33,69
	90–100	1,04	5,21	50,22	6,94	7,06	29,53
	100–110	0,66	3,92	54,83	6,90	6,07	27,62
Ckb	110–120	0,48	6,33	53,82	6,33	6,50	26,54

**Table 6:** Grain size distribution, mm (Laser granulometry).

Horizon	Depth, cm	0,25–1	0,05–0,25	0,01–0,05	0,005–0,01	0,001–0,005	<0,001
**Surface soil 1f-19**							
Аh	0–10	0	11,1	57,4	11,5	16,6	3,4
Ahe1	10–20	0	6,2	54,6	11,8	21,6	5,8
Ahe2	20–30	0	4,1	53,2	12,1	23,5	7,1
	30–40	0	3,2	54,0	12,5	23,0	7,3
AeB	40–50	0	4,7	54,8	11,6	21,2	7,7
Bt1	50–60	0	3,7	54,0	12,2	22,6	7,5
	60–70	0	5,0	52,9	11,4	21,8	8,9
	70–80	0	4,2	55,0	12,0	21,3	7,5
	80–90	0	3,3	52,5	12,8	23,3	8,1
Bt2	90–100	0	3,7	53,7	12,3	22,6	7,7
BCk	100–120	0	4,0	52,1	12,4	23,3	8,2
	120–140	0	2,4	43,8	12,3	30,4	11,1
	140–150	0	1,8	40,3	12,7	30,8	14,4
**Buried soil 2b-19**							
AhEb1	0–10	0	0,1	48,0	15,4	28,0	8,5
	10–20	0	0,1	49,5	15,4	26,6	8,4
AhEb2	20–30	0	0,2	50,6	14,9	25,9	8,4
AeBb	30–40	0	0,2	49,3	15,3	26,1	9,1
Btb	40–50	0	0,5	51,2	14,1	25,1	9,1
	50–60	0	0,4	50,6	14,8	25,1	9,1
	60–70	0,1	3,4	56,4	11,6	20,9	7,4
	70–80	2,6	4,0	53,9	11,3	20,4	7,2
BtCb	80–90	0	3,4	56,3	11,9	21,0	7,4
	90–100	0	2,4	54,9	12,5	22,3	7,9
	100–110	0	2,0	56,6	12,4	21,3	7,7
Ckb	110–120	0	1,3	53,6	12,3	23,0	9,8
	120–140	0	1,2	53,7	11,6	23,5	10,0
**Buried soil 3b-19**							
Ahb	0–10	0	2,8	52,4	13,1	24,3	7,4
	10–20	0	3,4	54,2	12,3	22,6	7,5
Aheb	20–30	0	3,9	57,0	11,8	20,5	6,8
AhBb	30–40	0	4,3	56,0	11,8	20,5	7,4
Btb	40–50	0	4,5	57,0	11,5	19,8	7,2
	50–60	0	4,1	55,3	12,2	21,0	7,4
	60–70	0	4,5	54,4	12,2	21,2	7,7
BCb	70–80	0	4,1	53,8	12,1	22,0	8,0
	80–90	0	3,9	52,5	12,5	22,7	8,4
	90–100	0	5,9	49,5	12,4	23,4	8,8
	100–120	0	5,0	48,0	12,7	24,9	9,4
Ckb	120–140	0	4,2	50,3	11,6	24,4	9,5
	140–150	0	4,5	49,7	11,7	24,4	9,7
**Buried soil 4b-19**							
Ahb	0–10	0	3,8	41,3	11,7	22,2	7,4
	10–20	0	3,2	53,9	12,0	23,5	7,4
	20–30	0	3,9	41,6	11,8	22,3	7,5
EBb	30–40	0	4,0	55,2	12,1	21,3	7,4
Btb	40–50	0	4,8	38,8	11,4	20,3	7,1
	50–60	0	4,4	55,7	11,6	20,9	7,4
	60–70	0	4,4	38,0	11,1	19,8	7,1
BtCb	70–80	0	4,7	54,1	12,4	21,2	7,6
	80–90	0	4,4	40,2	11,7	20,8	7,7
	90–100	0	7,2	60,3	9,7	16,7	6,1
Ckb	100–120	0	8,0	31,0	9,0	15,8	6,2
	120–140	0	4,9	51,8	10,4	23,3	9,6
**Buried soil 5b-19**							
Aheb	0–10	0	4,1	54,3	12,3	22,6	6,7
	10–20	0	3,6	55,5	12,4	21,7	6,8
AhEb	20–30	0	4,5	57,3	11,3	19,8	7,1
EBtb	30–40	0	4,9	58,0	11,2	19,2	6,7
Btb	40–50	0	3,9	55,9	12,0	20,8	7,4
	50–60	0	1,3	54,0	13,7	22,9	8,1
	60–70	0	0,6	51,0	15,1	24,8	8,5
BtCb	70–80	0	0,2	48,6	15,5	26,4	9,3
	80–90	0	4,7	53,4	12,1	21,8	8,0
	90–100	0	7,4	57,8	10,4	17,8	6,6
	100–110	0	7,8	59,0	9,3	17,0	6,9
Ckb	110–120	0	8,2	59,2	8,4	16,6	7,6

**Table 7:** Palynological spectrum for buried and surface soils.

Index	Pollen/ Spores/ Non-pollen palynomorphs	Soils						
		1f-19	2b-19 (1)	2b-19 (2)	3b-19	4b-19	5b-19 (1)	5b-19 (2)
P1	Pinus s/g Diploxylon	51,2	0,5	2	9,1	2,3	0	5,9
Ba	Betula sect. Albae	5,3	41,7	41,3	13,6	14,8	17,2	23,5
Al	Alnus	4,4	1,6	2,4	4,5	0,5	4,9	0
Sa	Salix	1,5	0	0	0	1	1,6	0
Q2	Quercus	7,1	1,1	0,8	0	3,1	0	0
U2	Ulmus	0,3	0,5	0,4	0	0	0	0
T2	Tilia	5,6	0,8	2	0	3,1	4,1	0
Cor	Corylus	0,2	0,8	0,4	0	1,3	0,8	0
Acer	Euonymos (Celastraceae)	0	0	0	0	0	0	0
Fr	Fraxinus	0,3	0	0	0	0	0	0
So	Sorbus	0	0,3	0	0	0	0	0
Ap	Apiaceae	0	0	0	0	0	0	0
Ar	Artemisia	2,3	6,3	3,1	0	2	3,3	0
At	Asteraceae	4,1	19,3	20,5	27,3	19,4	25,4	17,6
Xa	Xanthium strumarium	0,7	0,5	2,4	0	2	4,9	11,8
Cich	Cichorium	0	16,9	8,7	22,7	13	17,2	5,9
Crep	Crepis	0	0	0	0	0,5	0	0
Cc	Centaurea cyanus	0	0	0	0	0,3	0	0
	Cannabaceae	0	1,6	2,8	0	0,8	2,5	5,9
Br	Brassicaceae	0	0,5	1,6	0	16,9	0	0
Cr	Caryophyllaceae	0,2	0,5	1,6	9,1	0	0	0
Ch	Chenopodiaceae	3,6	0,5	0,8	0	1,8	0	5,9
Cy	Cyperaceae	2,1	1,6	0,8	0	1	0	5,9
Er	Ericales	0,2	0,3	0	0	0	0,8	0
Cal	Calluna vulgaris	0	0	0	0	0,3	0	0
Eu	Euphorbiaceae	0	0,3	0	0	0	0	0
Eph	Ephedra	0	0	0,8	0	0	0	0
Fa	Fabaceae	0,5	0	0	0	0	0	0
Po	Poaceae	3	2,1	5,5	13,6	11,5	8,2	11,8
Ро1	Poaceae (культурн.)	2,1	0,5	0	0	1,5	6,6	0
Pn	Polygonaceae	3,8	0,3	0,8	0	0,5	0,8	0
Pavic	P. aviculare	0	0	0	0	0,5	0	5,9
R	Rumex sp.	0	0	0,8	0	0	0	0
Pl	Polemoniaceae	0,2	0	0	0	0	0	0
Plantl	Plantago lanceolata	0	0,5	0	0	0	0	0
Plantst	P. stepposa	0,3	0,5	0	0	0	0	0
Gen	Gentianaceae	0	0	0	0	0	0	0
Lm	Lamiaceae (Labiatae)	0	0	0	0	0	0	0
Lin	Linaceae	0	0	0	0	0	0	0
Ma	Malvaceae	0,2	0	0	0	0	0	0
On	Onagraceae	0	0	0,8	0	0,8	0,8	0
Ra	Ranunculaceae	0,3	0	0	0	0	0	0
Tx	Taraxatum	0,2	0	0	0	0	0	0
RO1	Rosaceae	0	0,3	0	0	0,3	0	0
Pt	Potentilla	0	0	0	0	0	0	0
Sc	Scabiosa ochroleuca	0	0	0	0	0	0,8	0
Ru	Rubiaceae	0,2	0	0	0	0	0	0
U	Urticaceae	0,2	0,3	0	0	0,5	0	0
Val	Valerianaceae	0	0	0	0	0,3	0	0
Alis	Alismataceae	0,3	0	0	0	0	0	0
Lil	Liliaceae	0,5	0,3	0	0	0	0	0
Sp	Sparganium	0	0	0	0	0	0	0
Hych	Hydrocharitaceae	0	0	0	0	0	0	0
Ty	Typha latifolia	0,2	0	0	0	0	0	0
Tra	Trapa	0	0,3	0	0	0	0	0
Eq	Equisetum	0	0	0,4	0	0	0	0
Lcl	Lycopodium clavatum	0	0,5	0,8	0	0,3	0	0
Lcm	L. annotinum	2,1	1,1	6,3	18,2	1,8	4,1	0
Pd	Polypodiaceae	0,3	1,6	3,9	0	3,3	0,8	5,9
Pt	Pteridaceae	0	0	2	0	0	4,1	0
Bry	Bryales	0,7	1,6	0,4	0	0	0	0
Sph	Sphagnum	3,6	3,7	3,1	0	0,8	1,6	0
Ri	Riccia	0,5	0,3	2,4	4,5	1,3	2,5	0
An	Anthoceros	0,3	0	0,8	0	0,3	0	0
Alg	Algae (spherical)	1	0	19,7	9,1	3,8	5,7	5,9
Asco	Ascospores	0	0	0	0	0,5	0	0
Gsph	Geoglossum sphagnophilum	0	2,9	0	0	0	1,6	0
Br	Brachysporium bloxami	0	0	0	0	0	0	0
Gel	Gelasinospora	0	0,8	0,4	13,6	2,3	4,9	11,8
Gl	Glomus	0	2,1	0	0	1	4,1	0
Hpl	Helicoon pluriseptatum	0,3	0	0	0	0	0	0
M	Meliola ellisii, ascospore (parasite on Calluna vulgaris)	0	1,1	0	0	0	0	5,9
HdV88E	Microthyrium (HdV88E)	3,6	0	0	0	0	0	0
HdV182	HdV182	0	14,5	0	63,6	0	0	0
HdV463	HdV463	0,3	0	0	0	0	0	0
HdV16B	HdV16B	0	0	0	0	0	0,8	0
HdV265	HdV265	0	0	0	0	0	3,3	0
Mb	Macrobiotus	0	0	0	0	0	0	0
Pd	Podospora	0,7	0	0	4,5	1	0	47,1
Ps	Pseudoschizea	43,5	54,9	114,2	40,9	68,8	71,3	111,8
Puc	Puccinia-type	0	0,3	0	0	0	0	11,8
Sp	Sporomiella	3,1	0,5	0	0	0,3	0,8	41,2
Sord	Sordariaceae	1	2,9	0	0	0	2,5	0
Th	Thecaphora	0	0	0	0	0	0	0
	Testate amoeba	0	0,5	0	0	0	0	0
BM4	BM4 (Conidia)	0	2,6	0	0	0	0	0
Ust	Ustulina deustra	0	5,3	0,8	4,5	1	25,4	52,9
F und	Soil fungi udiff.	6,4	16,4	7,9	9,1	10,7	8,2	23,5
Z	Zygnemataceae	0	0	0	0	0,3	0	0
Xy	Xylomyces chlamidosporis/ aquaticus	0	0,5	0	0	0	0	0
E	Eggs	0,2	0,5	0	0	0	0	0
Charc	Charcoal particles	177,9	25,9	486,6	231,8	73,4	488,5	10,600
SUM(A)	Trees and shrubs	75,9	47,2	49,2	27,3	26,1	28,7	29,4
SUM(B)	Grasses ans herbs	24,1	52,8	50,8	72,7	73,9	71,3	70,6
SUM(C)	Aquatic and nearshore	1	0,5	0	0	0	0	0
SUM(F)	Ferns and mosses	7,6	8,7	20,1	22,7	7,7	13,1	5,9
SUM(N)	Non-pollen palynomorphs	60,1	105,8	142,9	145,5	89,8	128,7	311,8
SUM(P)	Charcoal particles	177,9	25,9	486,6	231,8	73,4	488,5	10,600
SSUM(SUM1)	AP	461	179	125	6	102	35	5
SSUM(SUM2)	NAP	146	200	129	16	289	87	12
SSUM(SUM3)	Aquatic	6	2	0	0	0	0	0
SSUM(SUM4)	Ferns and mosses	46	33	51	5	30	16	1
SSUM(SUM5)	Non-pollen palynomorphs	365	401	363	32	351	157	53
SSUM(SUM6)	Charcoal particles	1080	98	1236	51	287	596	1802
SSUM(S(SUM1))	AP+NAP	607	379	254	22	391	122	17

**Fig. 2:**
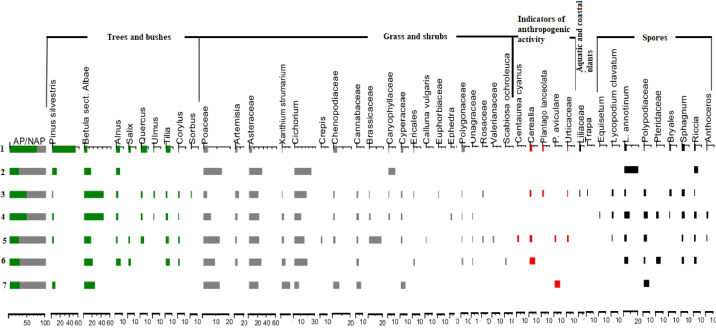
Spore-pollen diagram. 1- surface soil 1f-19, 2 – buried soil 3b-19, 3 - buried soil 2b-19 (1), 4 - buried soil 2b-19 (2), 5 - buried soil 4b-19, 6 - buried soil 5b-19 (1), 7 - buried soil 5b-19 (2).

**Fig. 3.**
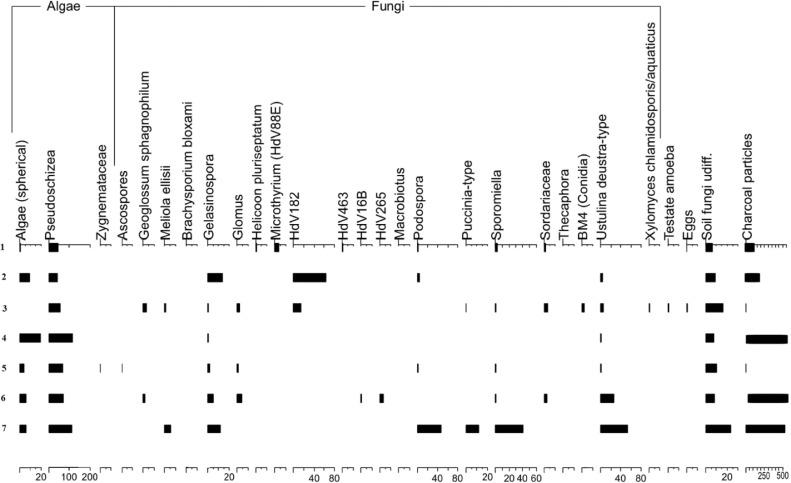
Diagram of non-pollen palynomorphs. 1- surface soil 1f-19, 2 – buried soil 3b-19, 3 - buried soil 2b-19 (1), 4 - buried soil 2b-19 (2), 5 - buried soil 4b-19, 6 - buried soil 5b-19 (1), 7 - buried soil 5b-19 (2).

Figs. 2 and 3 reflect data of Table 7. Fig. 2 shows spore-pollen diagram of the buried and surface soils. Non-pollen palynomorphs, such as charcoal particles, organic residues of aquatic microorganisms, spores of coprotrophic and parasitic fungi, and other indefinable spores of fungi are presented in Fig. 3.

## 2 Experimental design, materials, and methods

### 2.1 Dataset area and objects

Archaeologiacl excavations of the *Gochevsky* archaeological complex located on the right bank of the river Psel near settlement Gochevo, Belovsky district, Kursk region, were provided in 2019. The area is located within the Central Russian forest-steppe province of the East European Plain. *Gochevsky* burial ground contains funeral rites of the old Russian population of the southeast of Russia at the end of the X - XII centuries AD. All pits are located in a watershed surface under a forestland in Kursk region.

Soils buried under four mounds were examined. The soil profile can be considered as a data archive containing unique information about the features of past natural settings. All mounds were built in onetime interval but the difference between them is 25–50 years. The thickness of the mounds is 40–60 cm. The buried and surface soils were formed on the same topographical position, under the forestland and on the similar loess sediments. The distance between soils is ∼20 m. To compare the conditions of the past to the modern ones, the surface soil was also studied (1f-19). The soil 2b-19 was buried in the second half of XIth century, when the soils 3b-,4b-,5b-19 were buried in the second quarter/middle of XIth century. Samples for spore-pollen analysis from soil 2b-19 and 5b-19 were taken in two replicates due to fuzzy border of the buried soils. All soils were identified according to the WRB [3] as *Greyzemic Luvic Phaeozem Cutanic*.

### 2.2 Sampling and laboratory analysis

Samples from buried soils were collected below the burial embankment. Soil samples were taken from every 10 cm down to 1 m and 20 cm before 1 m but leaving the boundaries of the soil horizon intact. Samples for the spore-and-pollen analysis were collected from the upper 0–5 cm of buried and surface soils.

The soils were described according to the FAO Guidelines for Soil Description [1]. Soil colour was determined in the field using the Munsell Soil Colour Charts [2].

The total carbonate content was determined on the base of the destruction of CaCO_3_ by acid and subsequent precipitation of the carbonate ion [5] рН in water suspension (soil: water ratio 1:2.5) were analysed using standard methods [6]. Carbon and nitrogen content for C/N ratio were obtained using CHNS Elementar Analysensysteme. Dithionite and oxalate extractable fractions of iron were determined according to Mehra and Jackson [4].

Two methods to determine the granulometric composition of soils were used: pipette method, in which soil is dispersed by treating with a solution of sodium pyrophosphate (Na_4_P_6_O_18_) and laser granulometry method on the device “Analysette 22. Laser Klasse 1. Fritsch”*.*

For elemental analysis, ∼1 g of sample was dried in the oven at 105 °C. Samples were powdered, and mixed with a lithium tetraborate flux and then melted to produce a glass disc. The concentrations of major and trace elements were analysed by XRF after the determination of the loss on ignition (1000 °C).

The taxonomic identification of microfossils was carried out using published keys and atlases [7], [8], [9], as well as electronic databases of photo pollen and non-pollen palynomorphs (NPP database, Paldat, European pollen database, etc.) with a use of a Motic-B1- microscope 220A at magnification × 400. Non-pollen palynomorphs were additionally examined (Fig. 3): organic residues of aquatic microorganisms, spores of coprotrophic and parasitic fungi on decaying plants and roots of trees, and difficult and indefinable spores of fungi are combined into this group. In each sample, the number of coal microparticles, which are among the effective eco-indicators, was also calculated. To calculate percentage ratios and build spore-pollen diagrams, the Tilia/TiliaGraph/TGView software package [10,11] was used. In the percentage calculation, the sum of the pollen of trees and shrubs (AR) and herbaceous plants (NAP) - AP + NAP is taken as 100%. The percentage of all taxa is calculated from this amount.

## Appendix Supplementary materials

Image, application 1
